# Design, Bioanalytical, and Biomedical Applications of Aptamer-Based Hydrogels

**DOI:** 10.3389/fmed.2020.00456

**Published:** 2020-10-22

**Authors:** Ya Di, Ping Wang, Chunyan Li, Shufeng Xu, Qi Tian, Tong Wu, Yaling Tian, Liming Gao

**Affiliations:** ^1^Department of Respiratory Medicine, The First Hospital of Qinhuangdao, Qinhuangdao, China; ^2^Department of Respiratory Medicine, Chinese People's Liberation Army General Hospital, Beijing, China

**Keywords:** aptamer-based hydrogels, aptamer, hydrogels, bioanalysis, biomedicine

## Abstract

Aptamers are special types of single-stranded DNA generated by a process called systematic evolution of ligands by exponential enrichment (SELEX). Due to significant advances in the chemical synthesis and biotechnological production, aptamers have gained considerable attention as versatile building blocks for the next generation of soft materials. Hydrogels are high water-retainable materials with a three-dimensional (3D) polymeric network. Aptamers, as a vital element, have greatly expanded the applications of hydrogels. Due to their biocompatibility, selective binding, and molecular recognition, aptamer-based hydrogels can be utilized for bioanalytical and biomedical applications. In this review, we focus on the latest strategies of aptamer-based hydrogels in bioanalytical and biomedical applications. We begin this review with an overview of the underlying design principles for the construction of aptamer-based hydrogels. Next, we will discuss some bioanalytical and biomedical applications of aptamer-based hydrogel including biosensing, target capture and release, logic devices, gene and cancer therapy. Finally, the recent progress of aptamer-based hydrogels is discussed, along with challenges and future perspectives.

## Introduction

Hydrogels are a kind of high water-retainable material (containing up to 99 wt% water) with a three-dimensional (3D) polymeric network which is similar to natural tissue. Due to the hydrophilic residues in the backbone of polymers, an immense amount of water molecules are retained within their structures (1). In addition, an extremely large surface area with good porosity has abundant interior space for biomolecules to be retained within the system through Coulombic attraction (2), while maintaining their biological activities. Moreover, the polymers or polymer monomers are easily dissolved in water before crosslinking, while after crosslinking, they are in a gel state with a defined shape. Due to their excellent properties, hydrogels have attracted much attention over the past years as the elaborate scaffolds in drug delivery carriers (3), tissue engineering, sensors (4) and cancer therapy (5). Hydrophilic polymer networks of hydrogels are formed through the crosslinking of monomers or polymer chains via covalent bonds and/or non-covalent interactions including hydrogen bonding, electrostatic interactions, host-guest complexation and their combinations (6–11). Plus, hydrogels can be made from a very large range of building blocks including polymers, peptides (12–15), and surfactants (16), with different types, degrees of cross-linking, and properties leading to the nanoscopic structures, size range, physical properties, and functions of hydrogel (17). Great attention has been paid to explore the strategy to control the functionalities of hydrogels. For example, many of the stimuli-responsive hydrogels have been constructed by using polymers modified with specific functional units that can rapidly respond to external stimuli. A variety of physical and chemical changes of the hydrogel, including volume change and sol-gel transition, are particularly sensitive to specific external stimuli due to their component materials (18–21). These stimuli-responsive hydrogels have gained immense consideration because of their potential in drug delivery systems (22–26), sensors (27–32), cancer therapy (33–37), cell culture substrates (38–40), and tissue engineering (38, 41–44). Beyond these stimulants, many specific biomolecules, such as antibodies, nucleic acids (or DNA), and enzymes, that can rapidly respond to target analytes are used for functional materials to modify polymers in order to construct target-responsive hydrogels (45, 46).

Aptamers are used to construct polymer networks as the stimuli-responsive element in aptamer-based hydrogels, and due to their unique characteristics they have gained great attention among the development of hydrogels which are responsive to specific target analytes (45, 47). Nucleic acid aptamers are single-stranded DNA(ssDNA) or RNA molecules, commonly containing 12–80 nucleotides (48, 49), generated by the systematic evolution of ligands by exponential enrichment (SELEX) (50) from a random ssDNA or RNA library (usually 10^15^~10^16^ different sequences) by means of three main steps including selection, separation, and amplification. Aptamer DNA hybridization and aptamer-target recognition both have very high specificity. Aptamers also possess high recognition ability toward specific molecular targets including ions (51, 52), small molecules (53, 54), proteins (55, 56), and cells (57, 58), because aptamers fold into a unique secondary or tertiary structure to bind to a target of interest, depending on van der Waals forces, hydrogen bonds, or electrostatic interactions (48, 59–62). Since they were discovered in the 1990s by Tuerk and Gold (63) and Ellington and Szostak (50), aptamers have become smart, specific, and high-affinity probes in bioanalytical, diagnostic, and therapeutic applications. Moreover, compared to other antibodies, aptamers are often called chemical antibodies due to their unique properties: (1) Aptamers are structurally stable with little immunogenicity and are chemically synthesized using standard solid state phosphoramidite reactions, which minimizes the batch-to-batch variation and improves the reproducibility of hydrogel systems. (2) aptamers are highly selective and have an affinity to targets, and low-dissociation constant values (*K*_ds_, 1 × 10^−12^-1 × 10^−9^ M) (64), so that aptamers can specifically recognize and undergo changes of their substrates even at very low concentrations. (3) The molecular weight of an aptamer is between 5 and 20 kDa, which is smaller than antibodies (ca.150 kDa), leading to better tumor uptake kinetics (6). (4) Aptamers are stable in a wide range of temperature, solvents, and pH. (5) Aptamers can be synthesized by chemical or enzymatic procedures or by a combination of these two methods without any animal-based synthesis. (6) Aptamers are easily modified with other functional moieties and have the capability of directional amplification by polymerase chain reaction (PCR). These excellent characters of aptamers make aptamer-based hydrogels even more versatile, and are excellent components in hydrogel engineering: (1) Aptamers can chemically conjugate with polymers such as acrydite and carboxymethylcellulose to construct the hydrogel (65, 66). (2) Aptamers can be integrated onto the surface of particles by chemical or physical methods and mixed with a pre-gel solution to form particle/hydrogel composites (67, 68). (3) Aptamers can recognize both target molecules and trigger complementary sequences (69–72). Especially to trap or introduce drugs (73–75), nanoparticles (76–78) into aptamer-based hydrogels have greatly expanded the applications in biosensing, target capture and release, cell adhesion and targeted therapy.

**Scheme 1:**
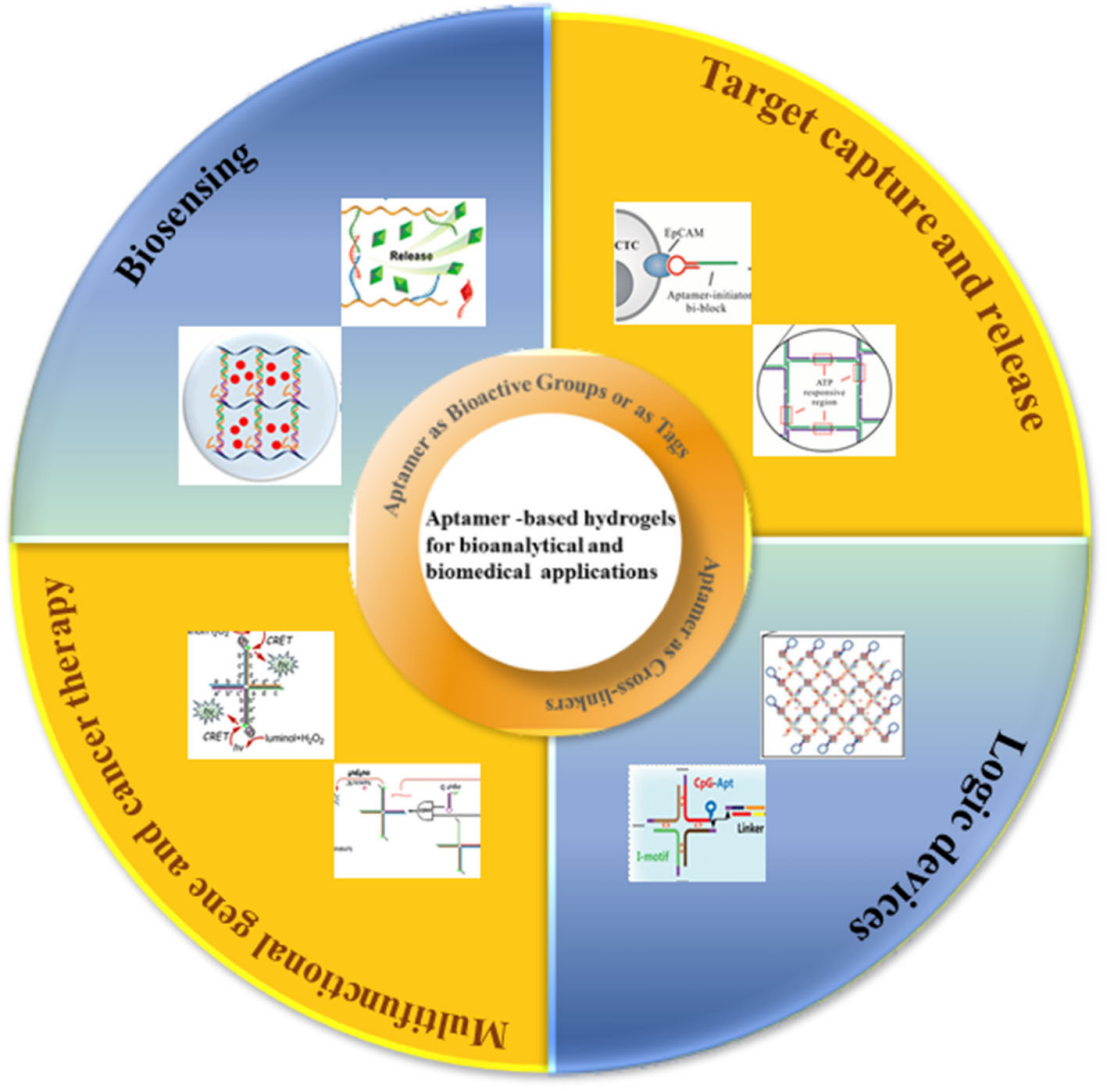
Schematic illustration of aptamer—based hydrogels for bioanalytical and biomedical applications.

In this review, we focus on the latest strategies of aptamer-based hydrogels in bioanalytical and biomedical applications (Scheme 1). We begin this review with an overview of the underlying design principles for the construction of aptamer-based hydrogels. Next, we will discuss some bioanalytical and biomedical applications of aptamer-based hydrogels including biosensing, target capture and release, logic devices, gene and cancer therapy. Finally, recent progress of aptamer-based hydrogels is discussed along with challenges and future perspectives.

## Design and Principle of Aptamer-Based Hydrogels

### Aptamer as Cross-Linkers

**Figure 1:**
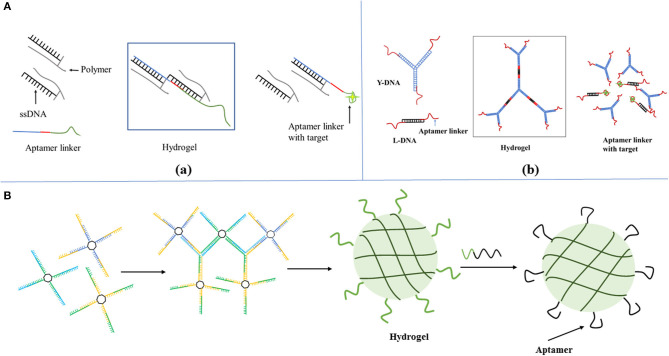
Schematic representation of the design strategy for formation and dissolution of aptamer-based hydrogel based on aptamers as **(A)** crosslinkers in (a) DNA functional polymer hydrogels [Reprinted with permission from Wang et al. (83). Copyright (2008) American Chemical Society] and (b) pure DNA hydrogels [Reprinted with permission from Previtera et al. (82). Copyright (2013) American Chemical Society]. **(B)** Bioactive groups or as tags for functionalization [Reprinted with permission from Lai et al. (84). Copyright (2019) American Chemical Society].

Aptamer-based hydrogels have been prepared based on different design principles. The selected design and preparation methods have a strong impact on the characteristic features of hydrogels and thus determine their respective biomedical applications. Using DNA aptamers as crosslinkers in hydrogels allows the hydrogels to be prepared to recognize the targets. In the absence of the target, the aptamer acts only as a conventional DNA crosslinker, but when the target is present, the aptamer preferentially forms a complex with the target and induces the change of the structure of the hydrogel. Such hydrogels utilize both the smart and programmable features of the DNA components as well as short aptamer sequences acting as supramolecular cross-linking agents (79–81). The first DNA-based polymer hydrogels were reported by Nagahara and Matsuda in which the short DNA sequences were grafted to a poly(acrylamide) polymer chain, and two pathways achieved gelation: (1) two DNA strands grafted to the polymer backbone were hybridized by other DNA sequences to induce the formation of gelation. (2) DNA strands attached to the polymer chain hybridized directly to form gelation without any external cross-linking agents (82). Based on this principle, a series of aptamer-based hydrogels have been prepared responding to target molecules (Figure 1Aa) (85, 86). Aptamers can also be used as crosslinkers in pure DNA hydrogels. A typical example is that a pure DNA hydrogel was constructed using a Y-shaped DNA and a thrombin aptamer linker through DNA self-assembly (Figure 1Ab) (87). The aptamers for ochratoxin A, ATP and adenine were used as DNA linkers to construct pure DNA hydrogels that were sensitive to targets (88–90).

### Aptamer as Bioactive Groups or as Tags for Functionalization

As we know, DNA segments as functional, bioactive elements rather than structural components were incorporated into hydrogels, and have also been explored for various bioanalytical and biomedical applications (84, 91–93). The presence of aptamer DNA as a bioactive group in these hydrogels typically does not change the mechanical properties and brings their high specificity toward a wide range of biological target molecules. Liu et al. (94) put forward a new strategy for fabricating a protein-scaffolded DNA nanohydrogel. By further incorporating therapeutic agents and tumor-targeting MUC1 aptamer, these SA-scaffolded DNA nanohydrogels can specifically target cancer cells and selectively release the preloaded therapeutic agents via a structure switching. A thrombin-binding aptamer was incorporated into the gel which can bind to adenosine, AMP, and ATP as shown in Figure 1B. DNA-functionalized gold nanoparticles or liposomes to DNA-functionalized hydrogels, when thrombin was added, a stable G-quadruplex structure emerged in the aptamer structure, which looked like a molecular switch between tight and relaxed states (95). X-shaped DNA, a DNA linker, and an aptamer were used to create a DNA hydrogel through the one-pot and the aptamer was only used as a functional unit for the target protein capture (96). A DNA nanohydrogel was developed to efficiently take up cells due to the recognition of an aptamer in the nanohydrogel (97). Moreover, an outstanding advantage of aptamer-functionalized hydrogels, as we know, would overcome the shortcomings of aptamers in bioanalytical and biomedical applications. Compared to antibodies, aptamers with high target-specific binding affinity values, were easily tailored for different targets. However, the drawback is their low cellular uptake for their high negative charge density and the limitation of stability in DNA degrading enzymes which are typically present in cells.

## Aptamer-Functionalized hydrogels for bioanalytical and Biomedical applications

### Aptamer-Based Hydrogels for Biosensing

Biosensors, as the powerful tools in monitoring biological or biochemical processes, have been applicated in various fields including medicine, disease diagnosis, food safety, and the environment (92). Nucleic acid aptamers are systematically engineered functional nucleic acids that demonstrate a very high affinity and specificity for targets including ions, metabolites, drugs, proteins, and even whole cells (98). Compared with antibodies, aptamers have been thought the ideal candidates as molecular recognition units to develop biosensors. Combined with DNA nanostructures possessing desirable advantages, aptamer-based biosensors hold great promise for the detection of a variety of targets. However, aptamers cannot freely penetrate the cell membrane and some nucleic acid probes are unstable in both intercellular and intracellular environments so that aptamer-based biosensors are usually compromised in intracellular environments (78, 99). Due to the protection of hydrogel nets, aptamer-based hydrogels have gained great attention in biosensing detection for their biocompatibility, chemical stability, and selective binding. Based on the designable conformational changes of aptamers, aptamer-based hydrogels can be combined with a variety of signaling mechanisms including fluorescence, electrochemistry, colorimetry, electroluminescence, and surface plasmon resonance, to construct rapid and sensitive biosensors to detect inorganic ions, organic small molecules, proteins, cells, and tissues. Usually, DNA-functionalized polymers have been simply crosslinked by the hybridization of aptamers with their complementary sequences to construct a hydrogel network structure. Because the binding affinities of aptamers to their target analytes are much stronger than that of simple hybridization, the network structure may deform or disintegrate when the specific target analytes are presented. The deformation of the aptamer-based hydrogel network can be easily detected with naked eyes or various colorimetric or fluorescence agents including, silver, gold nanoparticles, iodine, fluorescent dyes, and quantum dots (83, 100–107). The constructed hydrogel biosensor may achieve visual detection easily.

**Figure 2:**
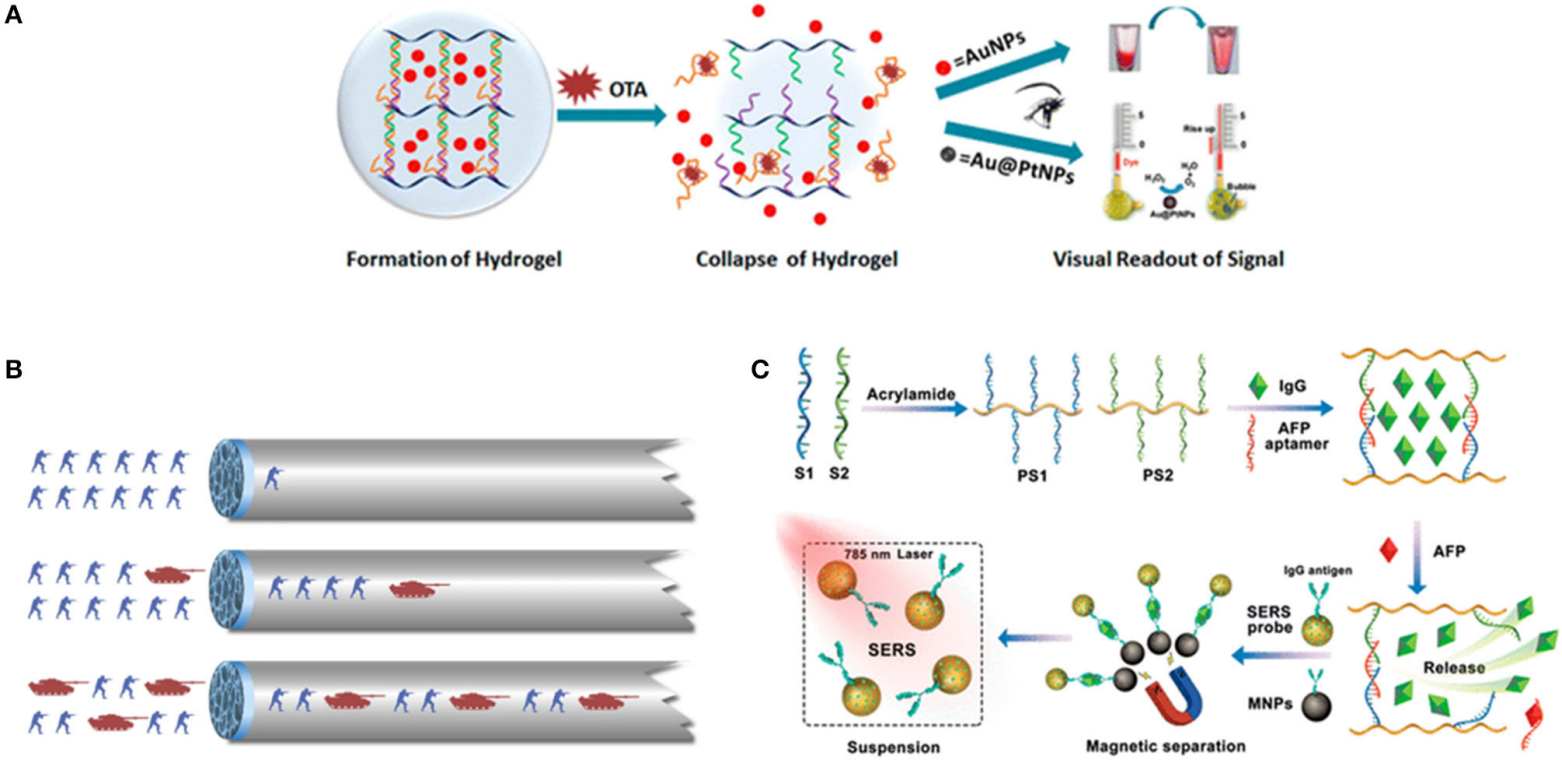
**(A)** Scheme of DNA-induced formation and adenosine-induced dissolution of hydrogel. Reprinted with permission from Wang et al. (83). Copyright (2008) American Chemical Society. **(B)** The scheme of a target-responsive hydrogel film in capillary tube for visual quantitative detection. [Reprinted with permission from Li et al. (110). Copyright (2019)]. **(C)** Preparation of aptamer functionalized hydrogels for the sensitive detection of α-fetoprotein using SERS method, Reprinted with permission from Guo et al. (103). Copyright (2020) American Chemical Society.

To detect biological molecules is vital for understanding their physiological and pathological functions. Since aptamers are easily modified and engineered, a large number of aptamer-based sensing hydrogel systems have been developed for the efficient detection of a wide range of biomolecules. Based on the use of DNA aptamers that cross-link with linear polyacrylamide chains, the first reported in this field was an aptamer-based hydrogel based on a gel-sol transition for detecting adenosine (85). In this design, when two oligonucleotide (DNA1, DNA2)-conjugated polyacrylamide chains (P1, P2) were mixed, a transparent and fluid state was obtained. Subsequently, upon the cross-linking of the oligonucleotides DNA3 was added, so that the above fluid system could undergo a sol-gel transition. The linker strand DNA3 contained three functional domains, that is, the complementary domains with DNA1, DNA2, and aptamer sequence domain. When target adenosine molecules were presented in system, aptamers competitively bound to adenosine molecules, leading to the breakdown of the hydrogel and target-responsive payload release. In order to achieve visual detection, gold nanoparticles were used as the indicator to add into the gel to monitor the process of gel-sol transition because of their unique optical properties. In the presence of adenosine, the upper buffer solution turned from colorless to red, indicating that the AuNPs had been released into the solution. The method was generally representative to use a target molecule as a trigger for the dissociation of the aptamer-based hydrogels to develop a biosensing system with high selectivity and visuality. In a similar work, a detection of food toxin, toxin A, was developed by applying this approach. The linear polyacrylamide polymers functionalized with short DNA strands were hybridized by OTA aptamer strands to construct the hydrogel network structure, and gold nanoparticles were still entrapped within the hydrogel using optical agent (108) (Figure 2A). An aptamer-functionalized DNA hydrogel was also prepared though DNA hybridization and incorporated inorganic nanomaterials including gold nanoparticles (AuNPs) and quantum dots (QDs) as signal indicators. The pure DNA hydrogel was directly constructed using Y-shaped DNA, linker DNA, and aptamer sequence with two different recognition sites for thrombin and the complementary sequence. Upon adding thrombin, it competitively bonded with aptamer, leading to the collapse and dissolution of the DNA hydrogel. The released negatively charged AuNPs would meet positively charged polyethyleneimine (PEI)-functionalized QDs and a fluorescence quenching strategy based on the Förster resonance energy transfer (FRET) was developed for the sensitive detection of thrombin in complex matrices (87). It is obvious that great progress has been made in this research area, however, regarding AuNPs, quantum dots etc. as visual indicators, there are several issues that need to be considered. For example, AuNPs exhibited an intense background color in the process of gel dissolution. As a result, this detecting method is not sensitive enough.

More recently, a thrombin-binding aptamer was incorporated into hydrogel. When thrombin was presented, a stable G-quadruplex structure in the aptamer structure emerged which changed the form of hydrogel by the molecular switch between tight and relaxed states (109). Li et al. (110) reported a gel film in a capillary tube based on the thermally reversible principle which transformed the analyte-induced small changes inside the DNA hydrogel into visual signals. In the analysis process, the permeability of the DNA hydrogel film will increase because of the small structural changes in the gel induced by the interaction between target molecules and the aptamer linkers, thereby changing the flow velocity of the sample solution in the capillary tube (Figure 2B). The duration time of the target solution flowing through the capillary tube with a specified length was used to characterize the concentration of different solutions. The ultra-trace aptamer DNA hydrogel (0.01 ml) detected cocaine directly with a low detection limitation (1.17 nM) and excellent selectivity as (Figure 1B). A novel SERS biosensing platform was constructed by combining the target-responsive DNA hydrogel for the sensitive detection of α-fetoprotein (AFP) (83). The aptamer as a linker strand in DNA hydrogel specifically recognized AFP and accurately controlled the release of immunoglobulin G (IgG) encapsulated in hydrogel. In the presence of AFP, the hydrogels were disentangled and the IgG was released. Interestingly, the released IgG was captured by SERS probes and bio-functional magnetic beads through the formation of sandwich-like structures to decrease the detecting signals, which significantly improved the detecting sensitivity (Figure 2C).

Furthermore, the exploration and introduction of new functional nanomaterials to produce aptamer-based hydrogel biosensing systems which are highly sensitive are also being developed. Aptamer-incorporated graphene oxide (GO) hydrogel without synthetic polymers was developed for the detection of antibiotics. GO hydrogels were readily prepared by physically mixing GO solution with adenosine. The fast gelation of the GO dispersion in the presence of adenosine would attribute to the strong hydrogen bonding and electrostatic interactions between the adenosine and the GO nanosheets. Aptamer chains flatly lay on the surfaces of GO sheets as a result of the strong π-π stacking interactions between the hexagonal cells of graphene and the ring structure of nucleobases in ssDNA, which had been elucidated as an effective driving force for assembling GO sheets into hydrogels (111, 112). Tan et al. (113) reported a fluorescence biosensor based on GO hydrogel incorporated with aptamers, which could selectively bind to tetracyclines. After GO hydrogels were formed, the fluorescence signal of fluorescence labeled aptamers was quenched for fluorescence resonance energy transfer (FRET). When hydrogel was exposed to the tetracycline, the fluorescence recovered. Using the quenching/recovering of fluorescence, this biosensor of GO hydrogel provided a quantitative analysis of tetracycline with high sensitivity at much lower concentration.

## Aptamer-Based hydrogels for Target Capture and Release

### Capture and Release of Circulating Tumor Cells (CTCs)

**Figure 3:**
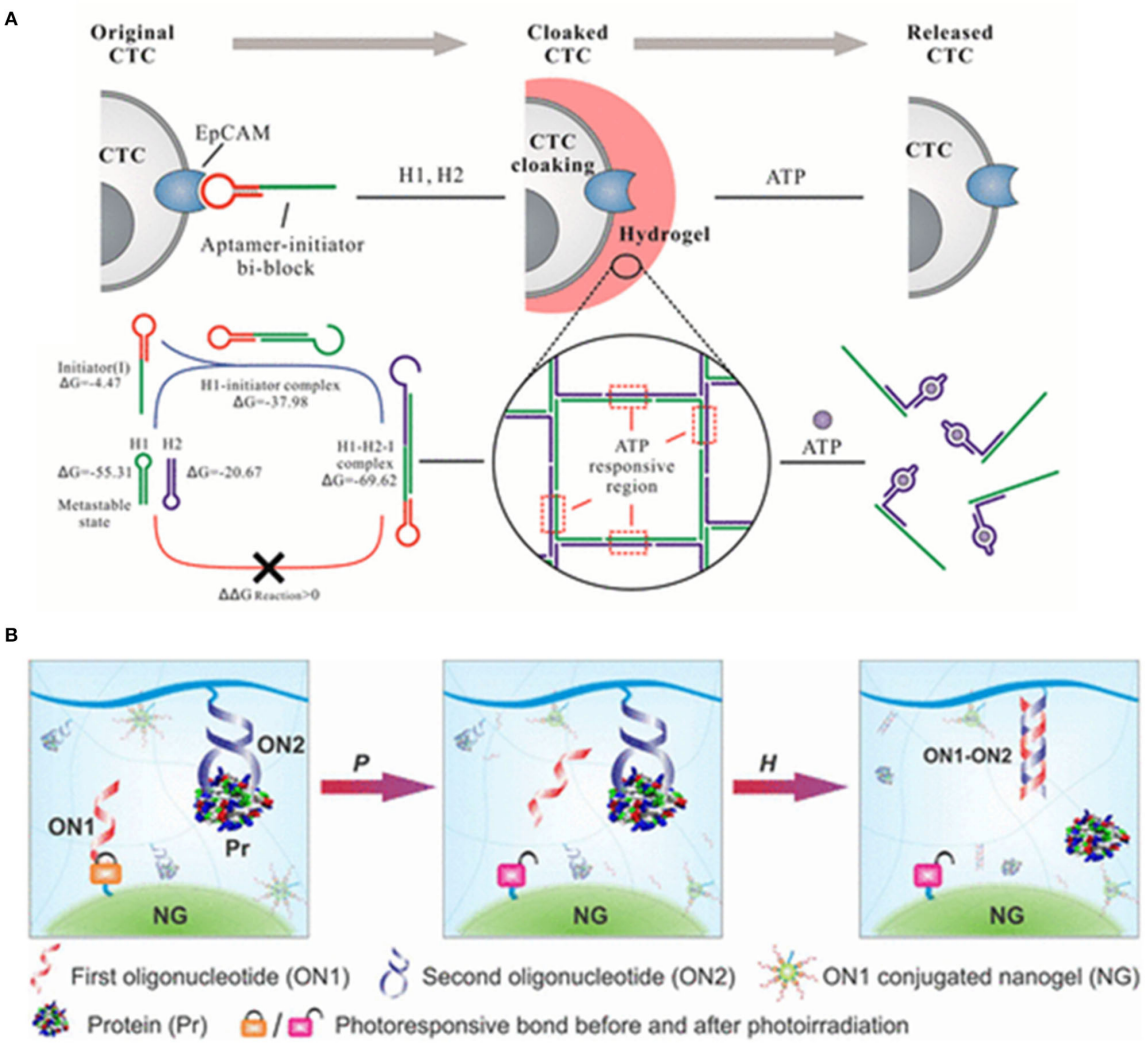
**(A)** DNA hydrogel with aptamer-toehold-based recognition, cloaking, and decloaking of circulating tumor cells for live cell analysis, reprinted with permission from Tan et al. (113). Copyright (2017) American Chemical Society. **(B)** Programmable self-assembly of protein-scaffolded dna nanohydrogels for tumor-targeted imaging and therapy, reprinted with permission from Pasparakis et al. (116). Copyright (2019) American Chemical Society.

Circulating tumor cells (CTCs) are the collective term for the tumor cells that escape from the primary tumor sites and travel through the circulatory system into the peripheral blood stream, at which point then, the metastases can be ultimately formed in resident organs. Therefore, detection of CTCs at early stages of tumors will increase diagnostic accuracy and therapeutic efficacy. However, CTCs are a very small population. In general, there are <10 CTCs/mL whereas there are approximately 5 × 10^9^ normal cells present in the same volume of blood sample (65, 114, 115). Therefore, a variety of materials have been recently investigated for sensitive catch and release of CTCs. Aptamer-based hydrogels as an emerging biomaterial, have recently attracted great attention in the fields of medical devices for cell catch and separation. For example, aptamer-based hydrogels were reported for *in situ* identification of live CTCs by cloaking/decloaking of CTCs (93). In this design as shown in Figure 3A, an aptamer DNA strand that specifically recognized epithelial cell adhesion molecule (EpCAM) on the CTCs surface triggered a hybridization chain reaction (HCR) via toehold-initiated branch migration. And an ATP aptamer was incorporated in the clamped HCR to decloak the DNA hydrogel on cell surface in order to achieve the phase transition from hydrogel to solution. The encapsulated AuNPs were exploited as the indicators of hydrogel formation via generating a red color at this state. Moreover, this method allowed to identify a low number of CTCs in whole blood by DNA hydrogel cloaking with high sensitivity and specificity for diagnosis. More significantly, controlled and defined chemical stimuli was used for the decloaking of CTCs without damages for subsequent culture and live cell analysis. Ye et al. (117) proposed an aptamer-trigger-clamped hybridization chain reaction (atcHCR) method for the capture of CTCs by porous 3D DNA hydrogels. The 3D environment of the DNA networks minimizes cell damage, and the CTCs can subsequently be released for live-cell analysis. In their work, initiator DNAs with aptamer-toehold biblocks specifically bind to the epithelial cell adhesion molecule (EpCAM) on the surface of CTCs triggering the atcHCR and the formation of a DNA hydrogel. The DNA hydrogel cloaks the CTCs, which would facilitate quantification with minimal cell damage. 10 MCF-7 cells in a 2-μl blood as sample were used to quantitively identify the decloaking of tumor cells via gentle chemical stimulus (ATP) which is used to release living tumor cells for subsequent cell culture and live-cell analysis. The whole experiment only was about 2.5 d including downstream cell culture and analysis. Aptamer-DNA hydrogels would open new powerful and effective routes for capturing rare live CTCs and their quantification in whole blood so that it can provide a new approach for cancer diagnostics and therapeutics. An aptamer-functionalized hydrogel was also reported that could catch CTCs with a density over 1,000 cells/mm^2^. When the hydrogel was coated by restriction endonucleases, the bound cells were released from the hydrogel coating because of the endonuclease-mediated sequence-specific hydrolysis of the aptamer sequences. The release efficiency reached 99%. Importantly, 98% of the released cells maintained viability (118). Polyvalent aptamer-functionalized hydrogel could also induce cell attachment on the hydrogel in dynamic flow. The cell density on the hydrogel was increased from 40 cells/mm^2^ to nearly 700 cells/mm^2^ when the shear stress was decreased from 0.05 to 0.005 Pa. After the attachment onto the hydrogel surface, approximately 95% of the cells could be triggered to detach within 20 min by using an oligonucleotide complementary sequence that displaced polyvalent aptamer strands from the hydrogel surface (119).

#### Capture and Release of Protein

To develop the efficient systems for the protection and sustained release of encapsulated molecules would be beneficial in improving how we treat disease and study complex biochemical processes. Exogenous signaling molecules as biochemical cues promoted mesenchymal stem cells (MSCs) survival, presumably because MSCs themselves can release a variety of potent signaling molecules. Zhao et al. (120) examined whether the release of exogenous signaling molecules from hydrogels can promote the survival of MSC spheroids. They thought that aptamer-functionalized fibrin hydrogel (aFn) could release exogenous VEGF and PDGF-BB in a sustained manner. PDGF-BB-loaded aFn could double the survival rate of MSC spheroids in comparison with VEGF-loaded aFn during the 1-week test *in vivo*. Therefore, aptamer-based hydrogels have been considered as the new and promising materials which could be used for encapsulating a variety of biomacromolecules because they are responsive to environmental changes and multiple stimulus could trigger conformational or chemical changes of elastic network in hydrogels resulting in deswelling or degrading of hydrogels. Aptamer-based hydrogels have been utilized as smart systems with sensitivities toward various non-invasive stimuli. However, several factors, including the pore size of the polymer network, the diffusion rate of the entrapped target molecule, and the affinity between the aptamer and target molecule usually influence the functionality and efficiency of aptamer-based hydrogels as capture-release systems (85, 116, 121–123). Moreover, biochemical signals or biomarkers stimuli are subtle or presented at subnanomolar concentrations. Therefore, a sensitive signal trigger is usually necessary to control the release of preloaded effectors in aptamer-based hydrogels (124–127). Recently, Lai et al. (128) developed new responsive hydrogels for controlled protein release by multistep molecular recognition events. Two oligonucleotides were integrated into the system as pendant motifs. The first oligonucleotide was used to covalently construct a hydrogel nanoparticle via a photolabile linker; and the other aptamer which could form a protein-DNA complex, was covalently conjugated to the bulk hydrogel network. When the hydrogel system was exposed to an external light signal, the nanogel was activated and dissociated. Subsequently, the freed oligonucleotide would hybridize with aptamer strands to induce the dissociation of the protein-DNA complex to release the bound protein (Figure 3B).

#### Capture and Release of Pollutants

An aptamer-based hydrogel was developed for water remediation with both high selectivity and multiple adsorbing abilities for several pollutants. In water remediation techniques, the contradiction between selectivity and multiple adsorptions limited this approach for environmental crisis previously (129). Aptamers in hydrogel were used to accommodate the molecular structure of pollutants in the scavenger and afforded the perfect selectivity. Meanwhile, Janus nanoparticles with an antibacterial function, in which aptamers were on the anisotropic surfaces to handle different kinds of pollutants. The final hydrogel scavenger was prepared by entrapping aptamer-functionalized Janus nanoparticles into a porous cellulose hydrogel. An aptamer column for the removal of trace pharmaceuticals in drinking water was reported (130). 5′-Aminomodified DNA aptamer bound to CNBr-Sepharose as sorbent was packed into gel as a column to simultaneously test cocaine and diclofenac in drinking water. The removal of pharmaceuticals was as high as 88–95%. The aptamer column was reusable and achieved a high removal efficiency from 4°C to 30°C in normal cation ion concentrations (5–100 mg L^−1^) for multiple pollutants without cross effects and secondary pollution.

### Aptamer-Based Hydrogels for Logic Devices

Nucleic acid molecules can be rationally designed, synthesized, and further integrated into Boolean operations, which provided an unprecedented potential to develop the basic components of molecular computing devices, because nucleic acid have high-capacity and low-maintenance digital information storage due to their predictable structures, high throughput synthesis and sequencing techniques (131, 132). Nucleic acid-based logic devices were first introduced in 1994 by Adleman and Lipton to solve the directed Hamiltonian path problem and the “SAT” question in computer science with single-stranded DNA sequences and enzymes (133, 134). Since then, science has seen the emergence of new logic systems for mimicking mathematical functions, diagnosing disease and even imitating biological systems (135–142). In recent years, logic gate systems based on aptamer-based hydrogels have attracted remarkable attention due to their intelligent responses to the external stimuli and convert input signals into a certain output signal.

Comparing this to silicon-based computation, although many challenges in designing computation devices, aptamer-based hydrogels logic circuits are still developing with great rapidity, due to their stability, biocompatibility, and predictable structure (143–146). Yin et al. (147) exploited a hydrogel structure based on hybridization behavior between crosslinker strands with aptamer sequences of ATP and cocaine molecules onto polymer chains. As detecting signals output, the BSA-modified gold nanoparticles were trapped in the hydrogel. The hydrogel served as an “AND” logic gate, when both cocaine and ATP presented, it was dissolved and led to the release of entrapped AuNPs And, the “OR” logic gate was reached if either cocaine or ATP presented, which led to the collapse of the hydrogel and release of the AuNPs. A novel colorimetric logic system based on an aptamer-crosslinked colloidal crystal hydrogel was also reported (148). When the Hg^2+^ and Ag^+^ responsive aptamers was incorporated into hydrogels, the reversible binding between the specific target ion (Hg^2+^ and Ag^+^) could induce the conformational change of the aptamers and thus make shrinkage of the hydrogels with different stimuli. The visualization of the logic output signals was realized, the aptamer-crosslinked hydrogel displayed a shrinking response and color change corresponding to a logical “OR” and “AND” gate when the stimuli of Hg^2+^ and Ag^+^ at a concentration of 0.1 μM was input.

**Figure 4:**
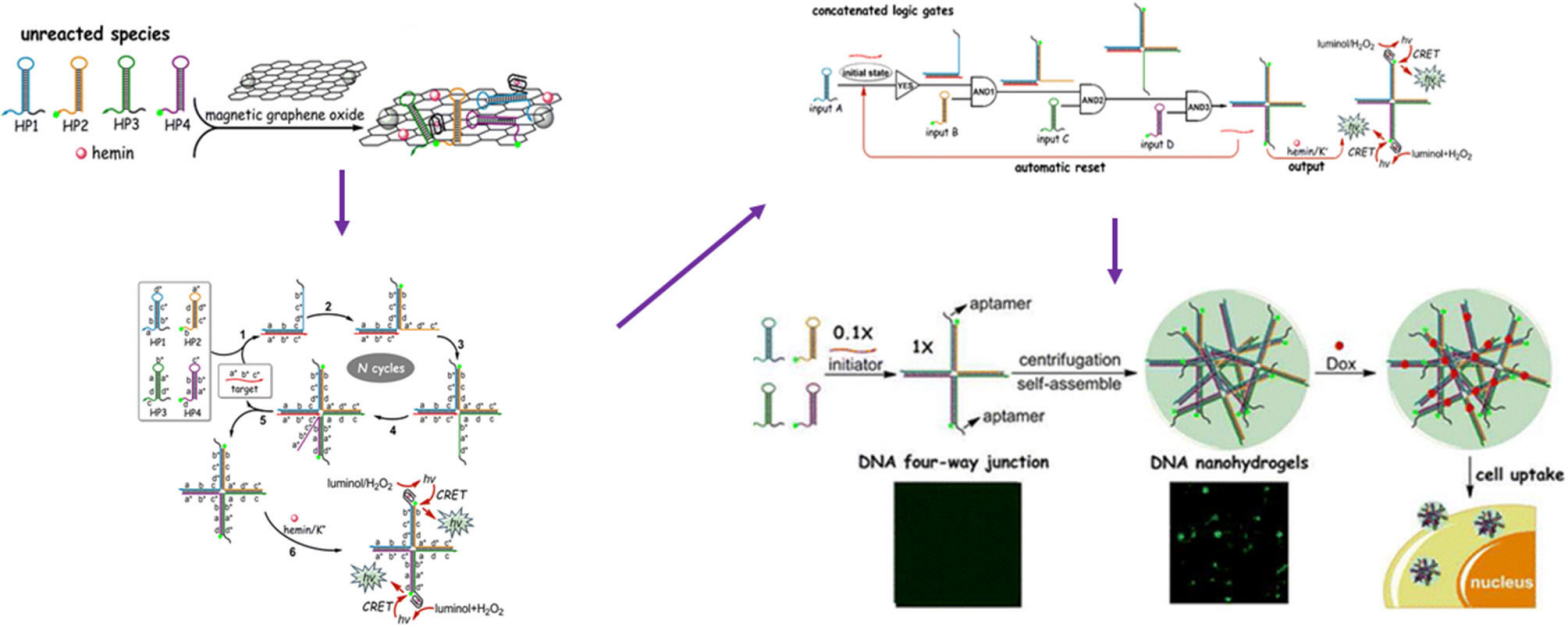
Schematic Illustration of the four-input concatenated logic gates based on target-catalyzed DNA four-way junctions. Reprinted with permission from Ramezani and Dietz (143). Copyright (2015) American Chemical Society.

Bi et al. (149) reported a DNA four-way junction (DNA-4WJ) which is target-catalytically formed through cascade assembly of four DNA hairpins on the basis of DNA TM-SDR. A concatenated logic circuit composed of one YES gate and three AND gates with an automatic reset function by using four DNA hairpins as inputs was fabricated and the formed DNA-4WJs serving as building units to construct DNA nanohydrogels (~120 nm). By incorporating aptamers, bioimaging agents, and drug loading sites into the building unit aptamer-based DNA nanohydrogels were synthesized with high loading capacity, target ability and good biocompatibility (Figure 4).

For traditional silicon circuitry, logic devices of aptamer-based hydrogels showed more powerful functions in medical diagnosis (150, 151), *in situ* analysis (151), and artificial intelligence. However, this is still in its infancy. Most of the reported works are conceptional, with isolated logic functions and limited applications. The breakthrough and development of novel design and construction might promote the development of logic devices of aptamer-based hydrogels.

### Aptamer-Based Hydrogels for Multifunctional Gene and Cancer Therapy

Gene therapy is a promising approach for the treatment of inherited diseases, such as cancers, hemophilia, and viral infections. It depends mainly on the research and development of the delivery vectors for gene. To achieve the safety and efficiency of gene delivery vectors, there remain many technical barriers to explore the potential of gene therapy. To date, gene therapy vectors mainly include viral vectors and non-viral vectors. Viral vectors are widely used for efficient gene transfer, but they are usually high-risk for immunogenicity and mutagenicity. In several clinical cases, their use has resulted in patient death. Compared with viral vectors, non-viral vectors are safer and more desirable. Therefore, the development of safe non-viral vectors is highly desirable (97, 149, 152, 153).

**Figure 5:**
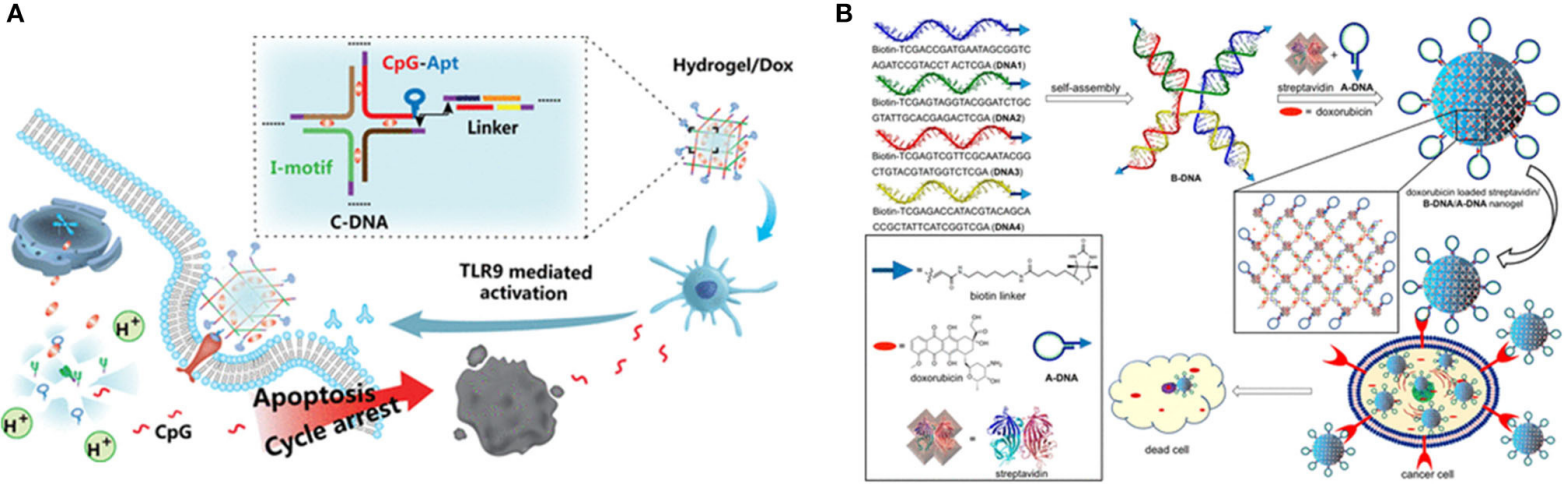
**(A)** Schematic diagram of the synthesis and action procedure of CpG-MUC1-hydrogel/Dox. Reprinted with permission from Ding et al. (168). Copyright (2019) American Chemical Society. **(B)** Doxorubicin-loaded nanogels using for delivery of doxorubicin. Reprinted with permission from Mazloumi Tabrizi et al. (154). Copyright (2019) American Chemical Society.

Recently, a variety of non-viral vectors, including liposomes (154), micelles (155), inorganic nanoparticles (156, 157), DNA nanostructures (73), and polymeric nano-hydrogel (158), have been explored as delivery vector for gene therapy. Among these, aptamer-based hydrogels are used as strong delivery vector candidates owing to their high payload capacity, as well as their biocompatibility, flexibility, and mechanical stability (78, 159, 160) as Table 1 shown. Tan (97) created a self-assembly process using three kinds of building units, Y-shaped monomer A with three sticky ends (YMA), Y-shaped monomer B with one sticky end (YMB), and DNA linker (LK) with two sticky ends, to hybridize a DNA nanohydrogel. By incorporating aptamers, disulfide linkages, and therapeutic genes into different building units, the aptamer-based DNA nanohydrogels (Y-gel-Apt) were formatted for targeted and stimuli-responsive gene therapy. And, a new intelligent DNA nano system integrating targeting, immunostimulant, and chemotherapy was also prepared based on unmethylated cytosine-phosphate-guanine oligonucleotides (CpG ODNs) DNA nanohydrogels (CpG-MUC1-hydrogel) (163). The cross-shaped DNAs (C-DNAs) assembled from pH-responsive I-motif sequences and targeted MUC1 aptamer-immunoadjuvant CpG-fused sequences (CpG-MUC1) were integrated into DNA nanohydrogels. DOX was successively intercalated into the base pairs of CpG-MUC1-hydrogel to form the CpG-MUC1-hydrogel/Dox that would controllably release DOX and CpGs at acidic conditions (Figure 5A). Moreover, a new class of physically cross-linked nanogel based on DNA, protein, and biotin as a nanocarrier using for the targeted cancer therapy was reported (162). The specific molecular recognition interaction between biotin and streptavidin was used to explore the cross-linking of a nanogel. The selective uptake of a doxorubicin-loaded nanogel by aptamer-receptor-positive cell lines (CCRF-CEM and HeLa) resulted in the specific interaction between the aptamer DNA decorated on the surface of the nanogel with the PTK7 receptor overexpressed on CCRF-CEM and HeLa cell lines (Figure 5B).

**Table 1:** A list of aptamer-based hydrogels for multifunctional gene and cancer therapy.

**Hydrogel materials**	**Aptamer function**	**Agents**	**Therapy application**	**References**
DNA	Bioactive Groups	Dox	On-demand drug release upon H2O2	(161)
DNA	Bioactive Groups	mRNA, MMP-9	GSH induce release of therapeutic genes	(97)
DNA	Bioactive Groups	Dox	Protonation triggering the release of the encapsulated drug	(162)
Polyacrylamide	Bioactive Groups	Dox	Near-infrared light-responsive drug delivery	(124)
DNA	Bioactive Groups	DOX, CpGs	pH induces transition of I-motif sequences	(163)
Polyacrylamide	Cross-linkers	Dox	Target protein nucleolin leads the gel to dissolve as a result of reducing the cross-linking density by competitive target-aptamer binding.	(164)
DNA, PLL-g-Dex	Cross-linkers	protein drugs	Complementary sequences (CSs) of aptamer induce release of protein	(165–167)
Carboxymethyl chitosan	Cross-linkers	Dox	ATP triggering sol–gel transition and DOX release	(166)

During clinical treatment, the side effects and accuracy of drug molecules in intravenous chemotherapy are the main topic of discussion for treatment. To design multifunctional therapeutic delivery nanoplatforms would overcome these limitations. A protein-scaffolded aptamer DNA nanohydrogel was fabricated by three types of streptavidin (SA)-based DNAtetrad accompanying with the further incorporation of therapeutic agents and tumor-targeting MUC1 aptamer. In an ATP-rich intracellular environment, this aptamer DNA nanohydrogel specifically targeted cancer cells and selectively released the preloaded therapeutic agents via a structure switching to attain the image and treatment of cancer cells (94). Furthermore, a novel class of physically cross-linked nanogels solely made of DNA, protein, and biotin were designed and the biotin–streptavidin molecular recognition interaction was used for the physical cross-linking of DNA nanostructures. Biotin-modified ssDNAs were assembled to form 5′-biotin-tethered X-shaped branched DNA acting as a tetravalent host and then streptavidin-modified aptamer DNAs interacted with them to form the aptamer-based hydrogels which allowed the loading of doxorubicin inside the gel network and delivered in the cancerous environment. The aptamer-functionalized and doxorubicin-loaded nanogels exhibited selective uptake into target cell lines (162). In the clinical treatment of tumors, the delivery of drugs or genes, nanogels nanocarriers need long term circulation in the blood, enhanced permeability and retention effect (EPR effect), enrichment, infiltration, uptake, and release of the drug or gene. Aptamer-based hydrogels as intelligent nanocarriers demonstrated excellent biocompatibility and high selectivity for target cancer cells (156, 169).

## Conclusions and Perspectives

In summary, the recent progress in preparing aptamer-based hydrogels has made these kinds of materials accessible for encouraging applications in bioanalytical and biomedical fields. Aptamer DNA as the unique building blocks have prompted the development in sensitive biosensors, drug delivery systems, and cellular scaffolds for regenerative therapies. In this review, we divided aptamer-based hydrogels into two categories according to the gelation mechanism: aptamer as cross-linkers, bioactive groups or as tags for Functionalization. Various synthetic strategies and applications have been detailed. It is worth noting that the aptamer technology enables the design of DNA hydrogels that can detect almost any type of analyte with high selectivity and sensitivity. New aptamers are easy to generate which would lead to continue to growth of addressable targets. Despite the tremendous progress in the development of aptamer-based hydrogels, several challenges remained: (1) Aptamer-based hydrogels, as DNA hydrogels have low storage modulus and consequent thixotropic property, in which the strength is much lower than of most conventional polymers. It is crucial to regulate the mechanical properties for biological applications (78). (2) Mechanism studies on synthesis and responsiveness of aptamer-based hydrogels are needed to promote the development of hydrogels in biosensing, controlled release, and tissue engineering. Although the release profile of some stimuli-responsive hydrogels has been investigated, theoretically kinetic studies are rarely reported to reveal the release characteristics of aptamer-based hydrogels. Deeper kinetic studies will promote the design of aptamer-based hydrogels for biological applications. (3) There are still some problems in large scale applications of aptamer-based hydrogels because of their high cost and difficulty of preparation. To develop more techniques and novel synthetic methods to obtain more aptamer DNA should be mainly considered in order to reduce the cost. Furthermore, to explore new materials to hybrid with aptamer to construct multiple hydrogels is also a solution for cost concern.

## Author Contributions

YD: conceptualization and writing-original draft preparation. PW: conceptualization. CL: investigation. QT and TW: investigation and writing-original draft preparation. YT and LG: supervision. SX: writing-reviewing and Editing. All authors read and approved the final manuscript.

## Conflict of Interest

The authors declare that the research was conducted in the absence of any commercial or financial relationships that could be construed as a potential conflict of interest.
